# An unusual case of abdominal pain: psychogenic vomiting complicated by spontaneous pneumomediastinum

**DOI:** 10.1186/s12890-023-02459-8

**Published:** 2023-07-21

**Authors:** Decai Wang, Lizong Rao, Shuyun Xu, Biwen Mo

**Affiliations:** 1 Department of Respiratory and Critical Care Medicine, Key Laboratory of Pulmonary Diseases of Health Ministry, Key Site of National Clinical Research Center for Respiratory Disease, Tongji Hospital, Tongji Medical College, Huazhong University of Science and Technology, 1095 Jiefang Avenue, Wuhan, 430030 Hubei China; 2 Department of Respiratory and Critical Care Medicine, Guangxi Zhuang Autonomous Region Education Department Key Laboratory of Respiratory Diseases, Guangxi Health Commission Key Laboratory of Glucose and Lipid Metabolism Disorders, Second Affiliated Hospital of Guilin Medical University, 212 Renmin Avenue, Guilin, 541004 Guangxi China; 3 Key Laboratory of Respiratory Disease of Zhejiang Province, Department of Respiratory and Critical Care Medicine, Second Affiliated Hospital of Zhejiang University School of Medicine, Hangzhou, 310000 Zhejiang China

**Keywords:** Abdominal pain, Spontaneous pneumomediastinum, Psychogenic vomiting

## Abstract

**Background:**

Spontaneous pneumomediastinum (SPM) was defined by the appearance of free air in the mediastinum that was not preceded by trauma, surgery, or other medical procedures. Among the numerous manifestations of SPM, abdominal pain had seldom been described.

**Case presentation:**

A 25-year-old man presented to the emergency department with nausea, vomiting, and abdominal pain for 7 days. The presenting clinical features and the radiological results were suggestive of psychogenic vomiting with spontaneous pneumomediastinum in a patient who suffered from abdominal pain.

**Conclusions:**

The special feature of this case was the elucidation of a rare cause of abdominal pain, which should be differentiated in patients with vomiting combined with abdominal pain. The importance of this case was that its recognition may prevent unnecessary procedures to rule out or treat other causes of abdominal pain.

## Background

The first case series of SPM was published by Louis Hamman in 1939, and therefore the condition was called Hamman’s syndrome [1]. SPM was reported to occur in 1 of every 12,000 admissions and in up to 0.3% of patients admitted for asthma [2]. Previous studies had reported several precipitating factors in SPM, including nausea or vomiting, cough, physical exercise, inhalational drug abuse, smoke, and mechanical ventilation [3–5]. Psychogenic vomiting was defined as persistent vomiting without a physical etiology [6]. It had been described by numerous authors, with Oscar Hill providing an excellent summary of many of the diagnostic features and developmental issues, and was regarded as a distinct condition from the more commonly recognized vomiting that occurs in anorexia nervosa and bulimia nervosa [7]. The commonest presenting complaint was chest pain and dyspnea in patients with SPM [3, 8, 9], however, pneumomediastinum could be accompanied by pneumothorax or pneumoperitoneum in some cases [10]. Abdominal pain was a rare symptom of SPM. In this case, an adolescent who reported experiencing abdominal pain had interesting radiologic evidence of SPM, a complication of psychogenic vomiting.

## Case presentation

A 25-year-old man presented to the emergency department with nausea, vomiting, and abdominal pain for 7 days. He had no prior history of abdominal trauma or surgery and did not use recreational drugs. Otherwise, he did not sustain any chest trauma, undergo any vigorous exercise, or had a diving history. He had a past medical history of depression, for which antidepressants had been stopped for more than half a year. He was a smoker and had been drinking for 10 years. The patient gave informed consent for his case to be described.

During his hospitalization, the physical examination revealed his vital signs were within normal limits and that he was saturating at 99% on room air. The respiratory, cardiac, abdominal, and neurological examinations were unremarkable. He was 165 cm tall, had a normal body mass index, and exhibited no Marfanoid features or other clinical features of connective tissue disorders. The patient continued to vomit several times daily after admission to the medical ward.

**Fig. 1:**
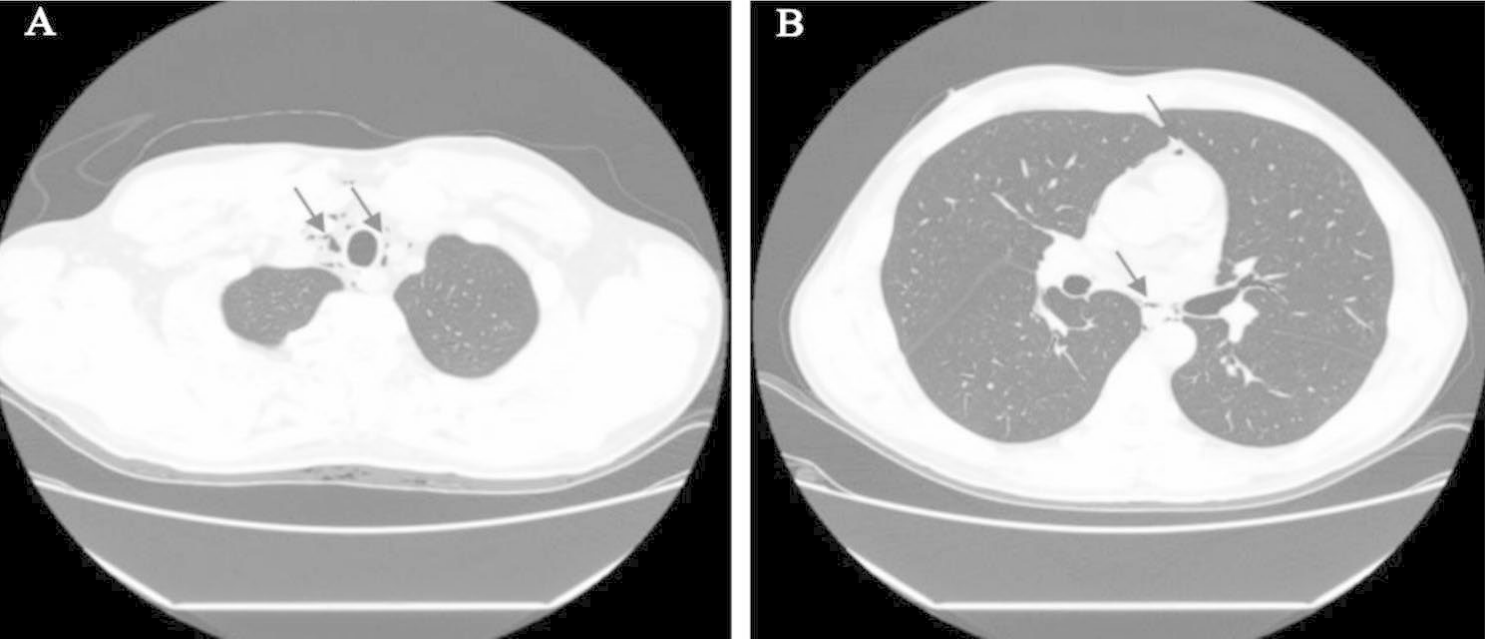
Chest Computed tomography-scan showed air streaks distributed from the paratracheal parenchyma to the pericardium (black arrow)

Extensive evaluations, including esophageal gastro-duodenoscopy, computed tomography (CT) of the abdomen, computed tomography angiography (CTA) of the abdominal aorta, computed tomography venography (CTV) of the mesenteric vein, brain magnetic resonance imaging (MRI), electroencephalography (EEG), electromyography (EMG), electrocardiogram (ECG), routine blood, examination of the stool for ova and parasites, serum amylase and liver enzyme studies, all failed to identify an organic source of his persistent vomiting and abdominal pain. However, as the black arrow shown in (Fig. 1). An unexpected pneumomediastinum was found by CT of the chest.

During his hospitalization, the patient was treated with bed rest, oxygen therapy, intravenous fluids, and antidepressions. Abdominal symptoms resolved after the correction of fluid and electrolyte disturbances. Pneumomediastinum resolved spontaneously and was followed up for 2 months without recurrence of any symptoms.

## Discussion and conclusions

The pathogenesis of SPM was thought to involve alveolar rupture secondary to increased pressure or overdistension, which leads to air dissection along perivascular and peripheral bronchial tissues up to the hilum of the mediastinum and to the soft tissues of the cervical region [11]. Of the potential precipitating causes of lung alveolar rupture, nausea or vomiting were the most common factors [12]. Smoking or vomiting could have been responsible for the SPM described in the above case. Although some studies had reported an association between SPM and smoking, the cause of SPM due to smoking was unknown [4, 5]. There were two special features of this case. Firstly, the cause of the vomiting was unknown. In patients with vomiting, important etiologies to consider were medication and toxin adverse effects, neurologic causes, gastrointestinal diseases, metabolic and endocrine conditions, and psychogenic disorders [13]. The patient had no prior medical history of neurologic disorders, metabolic and endocrine abnormalities, or usage of drugs or toxins. Psychogenic vomiting was a well-described clinical syndrome characterized by recurrent vomiting in the absence of demonstrable organic pathology [14]. Because the patient had a history of depression, the diagnosis of psychogenic vomiting complicated by spontaneous pneumomediastinum was made. What’s more, the reason for the abdominal pain was also uncertain. Acute abdominal pain was commonly caused by serious disease (e.g., vascular disorders such as aortic dissection and mesenteric ischemia), surgical conditions (e.g., appendicitis, cholecystitis), and abdominal wall problems (e.g., muscle strain or herpes zoster) [15]. The patient had no past history of abdominal trauma or surgery, and the abdominal examination, abdominal CT, CTA of the abdominal aorta, and CTV of the mesenteric vein revealed no abnormalities. Therefore, the abdominal pain might be caused by spontaneous pneumomediastinum following psychogenic vomiting.

Although chest CT was an important tool for detecting pneumomediastinum, pulmonary ultrasound was also used as a method of diagnosis for pneumomediastinum, or pneumothorax [16, 17]. The majority of pneumomediastinum patients got conservative treatment, which includes bed rest, oxygen therapy, and sufficient analgesia [18, 19]. The air was gradually absorbed from the mediastinum, and often administering a high concentration of oxygen allows the air to be absorbed more quickly [20]. Additionally, any underlying condition or cause should be properly addressed. In general, antibiotics were only used if a respiratory infection was the underlying cause or if there was any indication of mediastinitis [20]. If the pneumomediastinum was accompanied by a pneumothorax, a chest tube may also be inserted depending on the extent of the pneumothorax. After discharge, the patient should be advised to seek medical assistance on recurrent chest pain and shortness of breath. They should also be advised to refrain from vigorous activity and Valsalva maneuvers for the upcoming several weeks.

In conclusion, the unique aspect of this case was the identification of an uncommon cause of abdominal pain that should be distinguished in patients who have vomiting and abdominal discomfort. The significance of this case was that it may help to avoid needless operations to rule out or treat other causes of abdominal pain.

## Data Availability

The datasets used and/or analyzed during the current study are available from the corresponding author on reasonable request.

## Abbreviations

SPM: Spontaneous pneumomediastinum
CT: Computed tomography
CTA: Computed tomography angiography
CTV: Computed tomography venography
MRI: Magnetic resonance imaging
EEG: Electroencephalography
EMG: Electromyography
ECG: Electrocardiogram.
